# Aberrant Lighting Causes Anxiety-like Behavior in Mice but Curcumin Ameliorates the Symptoms

**DOI:** 10.3390/ani11092590

**Published:** 2021-09-03

**Authors:** Dhondup Namgyal, Kumari Chandan, Sher Ali, Ajaz Ahmad, Maha J. Hashim, Maryam Sarwat

**Affiliations:** 1 Amity Institute of Neuropsychology and Neuroscience, Amity University, Noida 201303, India; dhonamdhonam@gmail.com; 2 Amity Institute of Pharmacy, Amity University, Noida 201303, India; kumarichandandu@gmail.com; 3 School of Basic Sciences and Research, Department of Life Sciences, Sharda University, Greater Noida 201310, India; sher.ali@sharda.ac.in; 4 Department of Clinical Pharmacy, College of Pharmacy, King Saud University, Riyadh 11451, Saudi Arabia; aajaz@ksu.edu.sa; 5 Department of Life Sciences, University of Nottingham, University Park, Nottingham NG7 2RD, UK; mahajalal_73@yahoo.com

**Keywords:** curcumin, dim light at night, complete darkness, anxiety, weight gain, *PER1*, metabolism

## Abstract

**Simple Summary:**

In the present study, we exposed mice to aberrant lighting system and noticed anxiety-like behavior. These symptoms were ameliorated by oral administration of curcumin. The study was carried out on the animals for three weeks in dim light at night (dLAN) and complete darkness (DD), monitoring the body weight, daily food intake, anxiety-like behavior, and expression of the period (*PER1*) gene. The exposure to dim light at night was found to significantly enhance the anxiety-like behavior and increased the body weight possibly through altered metabolism in mice. In contrast, exposure to DD caused increased anxiety but no significant difference in the body weight. Moreover, the expression of the *PER1* gene involved in sleep was also found to be decreased in the aberrant light conditions (dLAN and DD). Although the treatment of curcumin had no effect on the body weight, it had ameliorated the anxiety-like behavior possibly by modulating the expression of the *PER1* gene. Thus, the alteration in the light/dark cycle has negative influences on body weight, affecting even the emotional quotient. This study identifies the risk factors associated with aberrant lighting conditions in laboratory animal and ameliorative effects of curcumin.

**Abstract:**

In the modern research field, laboratory animals are constantly kept under artificial lighting conditions. However, recent studies have shown the effect of artificial light on animal behavior and metabolism. In the present study on mice, following three weeks of housing in dim light at night (dLAN; 5lux) and complete darkness (DD; 0lux), we monitored the effect on body weight, daily food intake, anxiety-like behavior by employing the open field test, and expression of the period (*PER1*) gene. We also studied the effect of oral administration of different concentrations of curcumin (50, 100, and 150 mg/kg) for three weeks in the same mice and monitored these parameters. The exposure to dLAN had significantly increased the anxiety-like behavior and body weight possibly through the altered metabolism in mice, whereas exposure to DD caused increased anxiety but no significant difference in weight gain. Moreover, the expression of the *PER1* gene involved in sleep was also found to be decreased in the aberrant light conditions (dLAN and DD). Although the treatment of curcumin had no effect on body weight, it ameliorated the anxiety-like behavior possibly by modulating the expression of the *PER1* gene. Thus, alteration in the light/dark cycle had a negative effect on laboratory animals on the body weight and emotions of animals. The present study identifies the risk factors associated with artificial lighting systems on the behavior of laboratory animals and the ameliorative effects of curcumin, with a focus on anxiety-like behavior.

## 1. Introduction

The environmental lighting modulates several physiological and behavioral processes such as the sleep-wake cycle [1], blood pressure [2], hormone secretion [3], body temperature [4], and organ activities [5]. Nowadays, mankind has illuminated the nocturnal environment and adopted the artificial lighting system [6]. This has created overwhelming pollution, making natural celestial light almost unavailable to laboratory animals [7,8]. Studies on animal models have shown that artificial lighting results in circadian disturbances, inducing anxiety-like behaviors [9,10,11,12,13,14,15]. Moreover, the effect of the artificial lighting system is observed not only in laboratory animals but also poses a risk to human health. Recently, researchers reported that night shift workers have a higher risk of mood fluctuation and anxiety disorders [16,17,18]. Exposure to dim light at night (dLAN) has shown to raise various health issues such as disrupted metabolism, changes in immunological attributes, oxidative stress, sleep problems, and an alteration in circadian timing [19]. Similarly, the absence of light can be detrimental, especially in nocturnal animals. It can increase the active phase directly affecting the feeding and locomotor behavior of animals [20]. Thus, a better understanding of the effect of aberrant lighting on emotional regulations will enhance our understanding on the circadian rhythm and its associated attributes in laboratory animals. This may elucidate the underlying molecular mechanisms of behavior impairment faced in modern society and impel us to resolve these issues.

Earlier, we reported that exposure to dLAN and complete darkness (DD) for three weeks in Swiss Albino mice impaired both cognitive and non-cognitive behaviors through the modulation of hippocampal protein expression and that of core genes (*BDNF, CREB, DCX, SYN*, and *SIRT1*) associated with neurodegeneration [21]. Therefore, in this study, we undertook three weeks of exposure of mice to dLAN and DD to assess anxiety and altered homeostasis, together with the expressional change of the sleep gene *PER1*. Altering the light/dark cycle in nocturnal animals can either phase advance or phase delay the gene expression, which could lead to a disruption of metabolism and alteration of body mass. To assess the effects of the altered light/dark cycle on the metabolism and emotional/behavioral status of mice, we exposed them to artificial dim light (5 lux) at night and total darkness, monitoring the activity of the sleep gene period (*PER1*) in the hippocampus. The *PER1* gene is involved in multiple processes such as feeding behavior [22], including food anticipatory activity (FAA), sleep deprivation (SD), familial advanced sleep-phase syndrome (FASP) [23], and vulnerability to depression [24]. For this study, we selected 5 lux of artificial dim light at night, which is nearly five-folds more intense than the natural moonlight. Furthermore, this is equivalent to the lighting condition in modern laboratories and animal houses [25] but different from the level of daytime light. 

The role of plant-derived herbal drugs in mental health has been extensively explored in the past [26]. In current research work, we tried to monitor the deleterious consequences of aberrant lighting on the emotional and metabolic profile of mice by administering curcumin. It is a natural polyphenol extract of *Curcuma longa* (turmeric), used widely as a herbal modulator molecule since the beginning of Ayurvedic medicine. Several researchers have reported the health benefit of curcumin in neurodegenerative disorders {Parkinson’s disease, Alzheimer’s disease, multiple sclerosis, Huntington’s disease, schizophrenia, and anxiety-like behavior [27,28,29,30,31]. However, information regarding the modulatory role of curcumin in combating the effects of aberrant lighting in mice is not available. We pursued our hypothesis regarding whether exposure to dLAN and DD had caused anxiety and disrupted normal metabolism, and if these consequences may be ameliorated by the treatment of different concentrations of curcumin.

## 2. Materials and Methods 

### 2.1. Animals

A total of 45 Swiss Albino mice (either sex, 4 weeks old; weighing 25–30 g) were procured from the animal house facility of Amity University, Noida. The animals were kept individually in clean cages at the temperature 22 ± 2.4 °C. For sustenance, they were fed with Harlan Tekla 8640 food (Madison, WI, USA) and filtered water ad libitum. During the experimentation, mice were kept under the normal standard light and dark cycle (light/dark; {~150 lux/0 lux}) unless specified. The light intensity was measured regularly with a lux meter. All the experimental procedures of this study were duly ratified by the Institutional Animal Care and Ethical Committee of Amity University (CPCSEA/IA/EC/AIP/2017/03/02). The recommendations cited by the Committee for Control and Supervision of Experiments and Animals (CPCSEA) were used as guidelines for maintaining the mice during this period. Mice were weighed on the first day of the experimentation and then their weight was recorded every week. The total food intake was recorded daily (at 24 h interval) throughout the study period. 

### 2.2. Drugs and Biochemical Reagents

The curcumin (90% pure) used for this study was purchased from Sisco Research Laboratory (SRL) Pvt. Ltd., (Mumbai, Maharashtra, India). The High Capacity cDNA kit and phosphate buffer saline (PBS) were purchased from Thermo Fisher Scientific (Waltham, MA, USA). The total RNA isolation kit (Sigma Aldrich, St. Louis, MO, USA) and SYBR Green master mix (Applied Biosystems, Foster City, CA, USA) were used for the experimentation. Tissue homogenizer from Polytron (Thomas Scientific, Swedesboro, NJ, USA), NanoDrop (NanoDrop Technologies, Wilmington, DE, USA), and the StepOne Real-Time PCR System (Applied Biosystems, Foster City, CA, USA) were used for the present study.

### 2.3. Drug Preparation and Experiment Design

The different concentrations of curcumin were prepared by dissolving it in 1% carboxymethyl cellulose (CMC). The mice were divided into 9 groups (*n* = 5 per group) as given here.

LD group (1% CMC) (*n* = 5) (1)

dLAN group (1% CMC) (*n* = 5) (2)

DD group (1% CMC) (*n* = 5) (3)

dLAN + Cur50 (Curcumin 50 mg/kg) (*n* = 5) (4)

dLAN + Cur100 (Curcumin 100 mg/kg) (*n* = 5) (5)

dLAN + Cur150 (Curcumin 150 mg/kg) (*n* = 5) (6)

DD + Cur50 (Curcumin 50 mg/kg) (*n* = 5) (7)

DD + Cur100 (Curcumin 100 mg/kg) (*n* = 5) (8)

DD + Cur150 (Curcumin 150 mg/kg) (*n* = 5) (9)

Carboxymethyl cellulose (CMC) and curcumin were administered orally to the four-week-old mice once a day for three weeks. The experimental condition and details of treatment are depicted in Table 1.

## 3. Behavioral Studies

### 3.1. Open Field Test

The apparatus of the open field test consists of a square arena (40 cm × 40 cm) split into 4 center and 16 outer squares. The apparatus was kept under a medially lit room (~20 lux) and a video recording device was kept above the square arena for documentation and observation purposes. During the trial period, mice were kept at the center of the apparatus and were free to explore the arena for 5 min. However, during the final test, the mice were allowed to explore the test apparatus for 10 min. The behavior was observed through video recording. These behaviors reflected the stress and anxiety level of rodents.

(1) Percentage of freezing time (percentage of times the animal stands still in one place).
(2) Frequency of grooming (total number of times animals scratch and lick their body without motion).
(3) Frequency of changes in body posture (total number of times animals exhibit elongated body posture).

After each test, the test apparatus was wiped with ethanol (10%) to remove any odors of the previous mouse [32]. 

### 3.2. Brain Tissue Sample Collection

When the experimental duration was completed, the mice were anesthetized (with sodium thiopental (50 mg/kg)) and sacrificed. Tissue collection was done in the morning from 9:00 to 11:00 am. The hippocampus was dissected out on ice-cold surgical plates and preserved in liquid nitrogen for further study including RNA isolation and quantitative analysis of the sleep gene.

### 3.3. Total RNA Extraction and cDNA Synthesis 

From the frozen hippocampal tissue sample, the total RNA was extracted by the protocol described by Sarwat and Naqvi [33]. The RNA was dissolved in 30 µL nuclease-free water and quantified by NanoDrop (NanoDrop Technologies, Wilmington, DC, USA). For quantitative analysis, the relative RNA concentration was calculated at 260 nm and purity assessment was recorded at 260 nm/280 nm of the absorbance values. Moreover, RNA was analyzed by using 1% agarose gel electrophoresis for qualitative analysis. The cDNA was synthesized by using the High-Capacity cDNA kit (Thermo Fisher, Waltham, MA, USA). In brief, the RNA was reverse transcribed following the manufacturer’s instructions wherein 1000 ng of total RNA and 10X RT primers were used (21). The synthesized cDNA was stored at −20 °C in a freezer for subsequent analyses.

### 3.4. Quantitative RT-PCR

To study the expression profile of the sleep gene *PER1* of the mice exposed to aberrant lighting (dLAN and DD), quantitative real-time PCR was used on the unexposed control (LD) group and the mice treated with different concentrations of curcumin. In each reaction mixture, 1µL (~50 ng) cDNA was used with gene-specific forward and reverse primers (Table 2), along with the SYBR Green master mix. A Step OneTM Real-Time PCR System (Applied Biosystems, Foster City, CA, USA) was used for the assessment of the samples. Along with the gene of interest, β-actin was run as a reference control to normalize gene expression. The reaction procedure entailed a time duration of 10 min at 950C, followed by 45 cycles at 950C (15 s) and 600C for 1 min. All the experiments were performed in triplicates, along with negative and positive controls. The expression fold change of *PER1* mRNA from different samples were calculated with reference to the normal control group based on the threshold cycle (CT) values by using the following formula: Relative Quantification (RQ) = 2^−ΔΔCT^. 

### 3.5. Statistical Analysis

To compare the result of the body weight, food intake, anxiety-like behavior, and hippocampal *PER1* gene expression, non-parametric One-way ANOVA and post hoc Dunnett’s test was employed. All the data were analyzed in GraphPad Prism-8 software (San Diego, CA, USA). The values of *p* < 0.05 represent a statistically significant difference amongst the groups. 

## 4. Results

### 4.1. Exposure to dLAN Increases Body Weight in Mice

**Figure 1:**
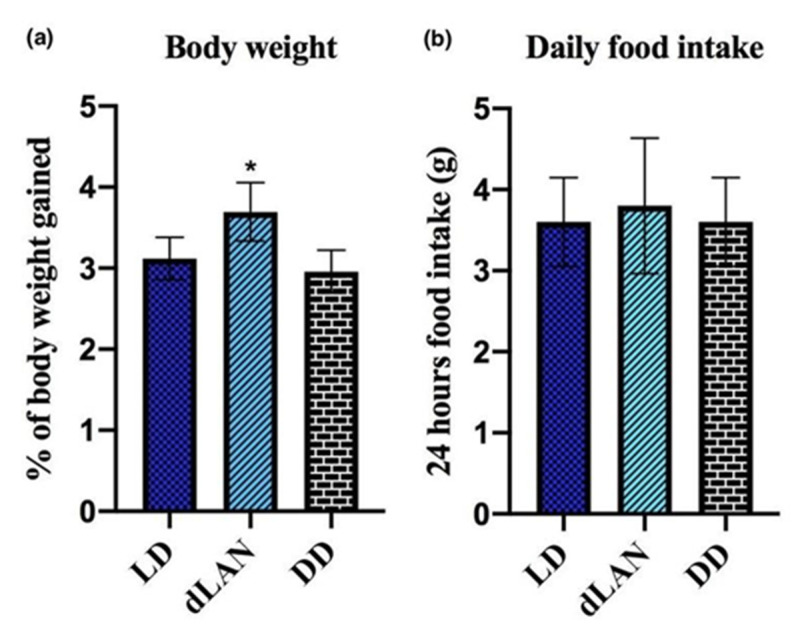
Effect of exposure to dim light at night (dLAN) and complete darkness (DD) on (**a**) the percentage of body weight gained and (**b**) the food intake. Values are represented as mean ± standard deviation (*n* = 5). Non-parametric one-way ANOVA with Dunnett’s post-hoc test to differentiate the results between the light/dark (LD) control vs. the dLAN control and DD control. All the data were analyzed with GraphPad Prism-8 software. The statistical values * *p* < 0.05 represent a significant difference among the groups.

Mice exposed to dLAN for three weeks gained significant body weight (F_2,12_ = 8.391, *p* < 0.05) compared to the normal LD control group (Figure 1a). This result suggests that dLAN exposure to nocturnal animals had increased the resting period by altering the sleep phase and thereby affecting their feeding and fasting behavior. This finding is supported by the result of DD-exposed mice as there is no significant difference in the percentage of weight gain between the mice exposed to DD (*p* > 0.05) and the LD control group (Figure 1a). As mice are nocturnal animals and they remain active during the dark period, exposure to DD had increased the active phase and thereby increased their metabolic rates. However, when we compared the daily food intake of mice in dLAN and DD groups against the LD control group, there was no significant difference amongst the groups (F_2,12_ = 0.1538, *p* > 0.05) (Figure 1b).

### 4.2. Curcumin Is Ineffective in Controlling Increased Body Weight Induced Due to dLAN Exposure 

**Figure 2:**
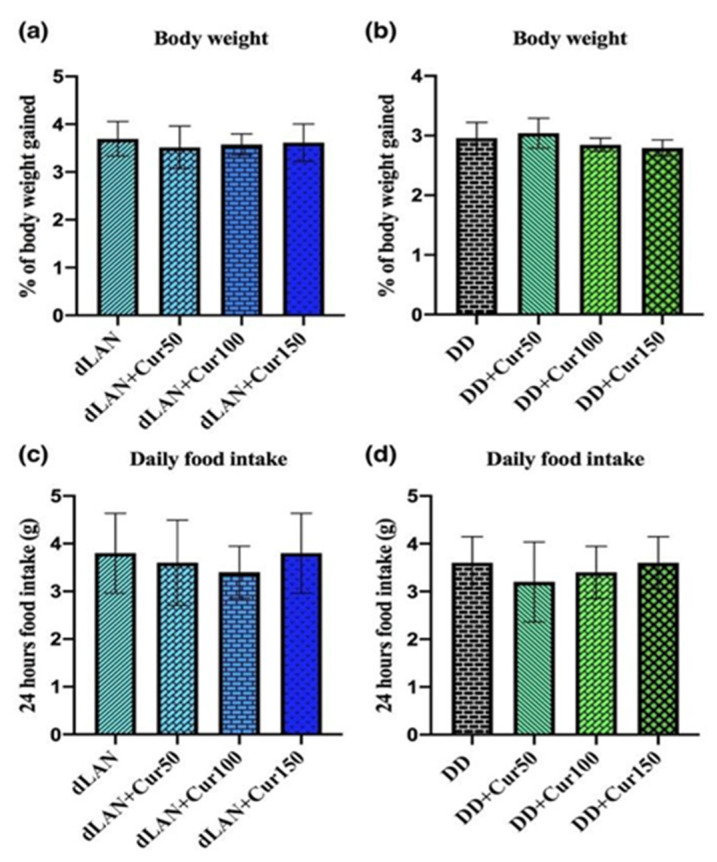
Effect of different concentrations (50, 100, and 150 mg/kg) of orally administered curcumin (Cur) on (**a**) the percentage of body weight gained in dim light at night (dLAN), (**b**) percentage of body weight gained in complete darkness (DD), (**c**) food intake in dLAN, and (**d**) food intake in DD-exposed mice. Values are represented as mean ± standard deviation (*n* = 5). Non-parametric one-way ANOVA with post-hoc Dunnett’s test was employed to differentiate the results among different treatment groups. All the data were analyzed with GraphPad Prism-8 software.

When the dLAN-exposed mice were treated with different concentrations of curcumin, we observed no significant difference in their increased body weight (F_3,16_ = 0.2111, *p* > 0.05) (Figure 2a) as compared to their normal vehicle control groups. The DD-exposed mice also did not show any significant difference in increased body weight (F_3,16_ = 1.536, *p* > 0.05) (Figure 2b) in comparison to their normal control groups. Curcumin is an herbal drug, which has pleiotropic medical benefits, but failed to produce a significant effect on correcting the increased weight gain. It also showed no effect on the daily food intake of mice exposed to dLAN (F_3,16_ = 0.2933, *p* > 0.05) (Figure 2c) and DD (F_3,16_ = 0.4583, *p* > 0.050.05) (Figure 2d) as compared to their respective normal vehicle control groups.

### 4.3. Aberrant Lighting (dLAN and DD) Caused Anxiety-like Behavior in Mice 

**Figure 3:**
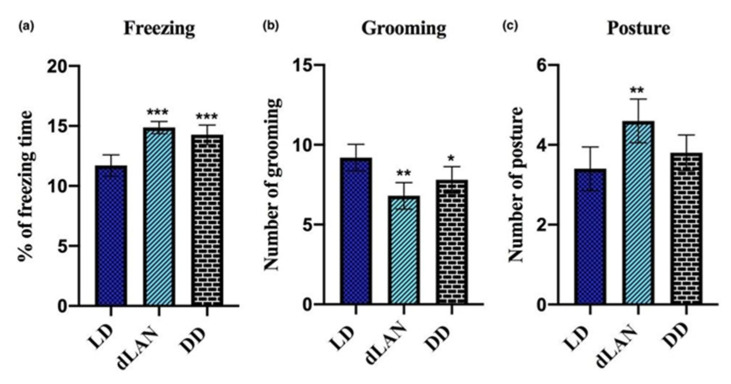
Effect of exposure to dim light at night (dLAN) and complete darkness (DD) on the anxiety-like behavior of mice. (**a**) Percentage of freezing time, (**b**) grooming, and (**c**) posture. Values are represented as mean ± standard deviation (*n* = 5). Non-parametric one-way ANOVA with post-hoc Dunnett’s test was employed to match the results between the light/dark (LD) control vs. the dLAN control and DD control. All the data were analyzed with GraphPad Prism-8 software. The statistical values * *p* < 0.05, ** *p* < 0.01, and *** *p* < 0.001 represent a significant difference among the groups.

Open field test results revealed that mice exposed to dLAN and DD for three weeks had higher anxiety and depression-like behavior as matched to the normal control group. The percentage of the freezing time is significantly higher in dLAN and DD-exposed mice (F_2,12_ = 24.96, *p* < 0.001) as compared to the normal LD group (Figure 3a). This was supported by the frequency of grooming as it is considerably decreased in dLAN (F_2,12_ = 10.38, *p* < 0.01) and DD (F_2,12_ = 10.38, *p* < 0.05)-exposed mice (Figure 3b). Moreover, the number of elongation postures was significantly increased in dLAN (F_2,12_ = 7.00, *p* < 0.01)-exposed mice (Figure 3c). These findings suggested that the dLAN-exposed mice are more prone to develop anxiety-like behavior.

### 4.4. Curcumin Improves the Anxiety-Like Behavior in Mice Exposed to dLAN and DD

**Figure 4:**
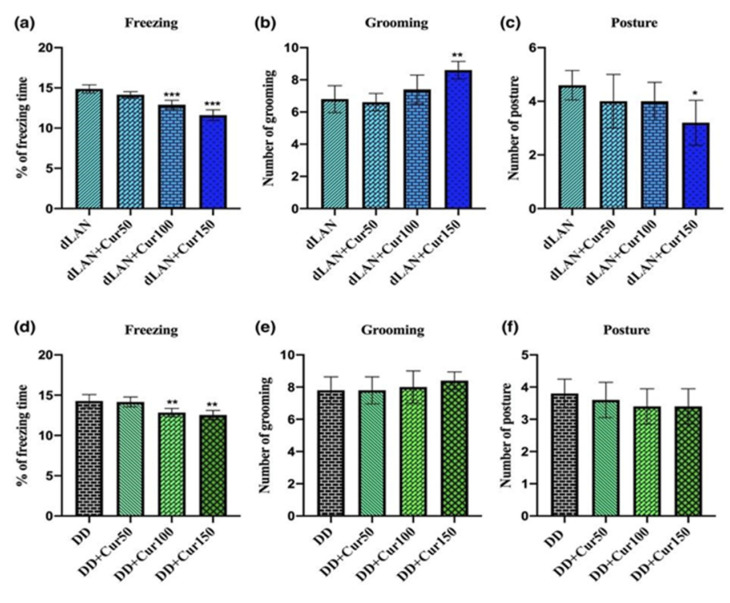
Effect of different concentrations (50, 100, and 150 mg/kg) of orally administered curcumin on anxiety-like behavior of mice. (**a**) Percentage of freezing time in dim light at night (dLAN), (**b**) grooming in dLAN, (**c**) posture in dLAN, (**d**) percentage of freezing time in complete darkness (DD), (**e**) grooming in DD, and (**f**) posture in DD-exposed mice. Values are represented as mean ± standard deviation (*n* = 5). Non-parametric one-way ANOVA with post-hoc Dunnett’s test was employed to match the results among different treatment groups. All the data were analyzed with GraphPad Prism-8 software. The statistical values * *p* < 0.05, ** *p* < 0.01, and *** *p* < 0.001 represent a significant difference among the groups.

However, the treatment of curcumin (150 mg/kg) had significantly reduced the percentage of freezing time in dLAN (F_3,16_ = 34.88, *p* < 0.001)-exposed mice (Figure 4a). In addition, curcumin (150 mg/kg) had increased the frequency of grooming in dLAN (F_3,16_ = 7.714, *p* < 0.01)-exposed mice (Figure 4b). Conversely, the treatment of curcumin (150mg/kg) reduced the elongation posture (F_3,16_ = 2.640, *p* < 0.05) of mice exposed to dLAN (Figure 4c). In the DD-exposed mice, curcumin treatment significantly increased the percentage of freezing time (Figure 4d). Furthermore, curcumin did not affect the grooming (Figure 4e) and elongation posture of mice (Figure 4f) exposed to DD. Therefore, these findings suggest that treatment of curcumin (150 mg/kg) could reverse the anxiety-like behavior induced by exposure to an altered light/dark cycle.

### 4.5. Influence of Aberrant Lighting (dLAN and DD) on the Hippocampal Sleep Gene PER1 

**Figure 5:**
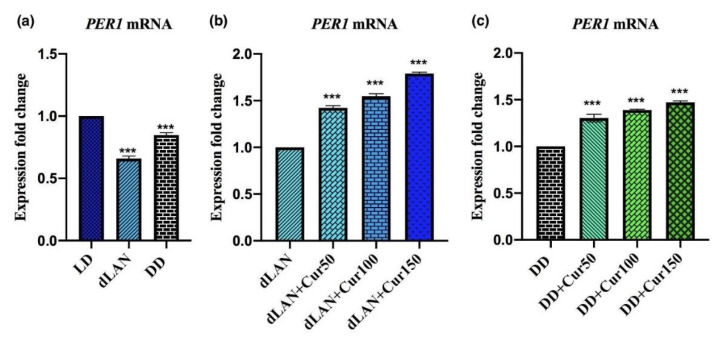
Effect of different concentrations (50, 100, and 150 mg/kg) of orally administered curcumin on the expression fold change of clock gene *PER1* in dim light at night (dLAN) and complete darkness (DD)-exposed mice. (**a**) Light/Dark (LD) control vs. dLAN and DD, (**b**) dLAN control vs. dLAN treatment, and (**c**) DD control vs. DD treatment. Values are represented as mean ± standard deviation (*n* = 5). One-way ANOVA with post-hoc Dunnett’s test was employed to match the results among the different treatment groups in different lighting conditions. All the data were analyzed with GraphPad Prism software. The statistical value *** p < 0.001 represent a significant difference among the groups.

To analyze our hypothesis that exposure to dLAN and DD for three weeks had a deleterious effect on the normal rhythmic expression of the sleep gene *PER1*, we assayed the diurnal expression of transcripts of *PER1* in the hippocampus of mice exposed to dLAN and DD for 3 weeks. The result indicates that exposure to dLAN (F_2,9_ = 472.5, *p* < 0.001) and DD (F_2,9_ = 472.5, *p* < 0.001) for three weeks had significantly decreased the expression of *PER1* mRNA (Figure 5a). This finding suggests that altering the normal light/dark cycle of mice by exposing them to dLAN and DD can modulate the rhythmic expression of the hippocampal sleep gene *PER1*.

### 4.6. Curcumin Increases the Expression of the Hippocampal Sleep Gene PER1 in Mice Exposed to dLAN and DD 

Moreover, treatment of various curcumin concentrations had significantly increased the expression of this gene in the mice exposed to dLAN (F_3,12_ = 1129, *p* < 0.001) (Figure 5b). An increase in the expression of *PER1* can also be seen in the DD-exposed mice when they are treated with varying doses of curcumin (F_3,12_ = 334.3, *p* < 0.001) (Figure 5c). These results suggest that the expression of the hippocampal *PER1* gene is under the control of the daily light/dark cycle and curcumin exerts its recuperation effect dose-dependently.

## 5. Discussion

The use of artificial nighttime light has become unavoidable in contemporary laboratories as the rate of urbanization and utilization of artificial lighting systems has been growing rapidly. Animals’ physiological processes are tuned to the external light/dark cycle of their surroundings. The effects of night-time light and constant darkness on the brain and behavior of laboratory animals are recent findings [21]. Recent studies show that chronic exposure to artificial light during the night elevates the chances of developing behavioral impairment in laboratory animals, such as sleep disturbance [34,35], mood fluctuation [36], and metabolic disorders [37]. The effect of aberrant lighting conditions is not only confined to laboratory animals, though, as the prevalence of stress and anxiety-related mood disorders are markedly greater in nighttime workers as matched to normal daytime workers [38]. These findings indicate that excessive exposure to the aberrant lighting conditions harm brain physiology and the behavior of laboratory animals [39]. 

Considering the external light/dark cycle synchronizes the circadian rhythm of organisms, any disturbance in the light/dark cycle has a direct implication towards the physiological and psychological state of animals. Moreover, the center clock genes in the SCN control and regulate different hormones in our body through the neuroendocrine system. Therefore, the fluctuation of the daily light/dark cycle plays an important part in the prevalence of stress and anxiety-like mood disorders [40]. In the current study, we observed that exposure to dLAN and DD for three weeks had drastically increased the stress and anxiety-like behavior of mice (based on the percentage of the freezing time and frequency of elongated body posture). Moreover, it also decreased the instinctive behavior of mice, which encompasses grooming. As mice are nocturnal animals, exposure to dLAN had a more deleterious effect on their behavior as compared to DD. However, the administration of curcumin (150mg/kg) had effectively restored some of the anxiety-like behaviors including the percentage freezing time and frequency of grooming in mice. Corroborating this finding, in our previous study, we observed that the administration of curcumin in the Cd-induced neurotoxic mice model and normal light/dark-exposed mice revealed increased locomotor activity, reduced stress and anxiety-like behavior, and improved spatial learning and retention memory through the promotion of hippocampal neurogenesis [21,41,42].

Furthermore, it is reported that prolonged exposure to nighttime light can cause metabolism-related disorders in animals [25,37,43]. In our study, we observed an increased the percentage of weight gain in mice exposed to dLAN, whereas there was no change in the DD-exposed mice and the LD control groups. Similarly, Fonken et al. [9] observed increased body mass (15 %) in mice that were kept under dLAN (5 lux) conditions when matched to mice kept under normal lighting conditions. Borniger et al. [44] also reported that when the dLAN and LD-exposed mice were compared, mice exposed to dLAN had a reduced expenditure of energy in the whole body and increased both body mass and carbohydrate oxidation over the fat oxidation. Further corroborating our results, Russart et al. [20] showed that dLAN exposure for 8 weeks resulted in glucose intolerance and insulin resistance in mice. They observed the effect to be reversed when the mice were returned to normal LD conditions. dLAN causes an increase in the susceptibility of mice for type 2 diabetes mellitus (DM). The survival chances were reduced in mice having DM when exposed to dLAN [20]. In our study, an increased percentage of weight gain was not reported in the DD-exposed mice. Corroborating our findings, other scientists have also reported long-term light deprivation (DD) to not affect the percentage of body weight gain or adrenal weight in nocturnal animals [45,46].

Now, the question arises, what could be the reason for the increased percentage of weight gain in dLAN-exposed mice? It may be through altered feeding habits, sleep, or metabolism. When we examined the metabolism, we could not find any difference across the studies involving various groups (dLAN, DD, and LD). This means that the metabolism of dLAN-exposed mice and sleeping behavior can be faulty, as the timing of the food intake changed in the nocturnal mice. Mice, being nocturnal animals, eat and remain active at night and do not eat and rest during the day. This is explained very well by Nelson and Chbeir [47] who compared this phenomenon in mice to a similar phenomenon in human beings called night eating syndrome or night-time hyperphagia. Other reports also showed equivalent feeding [44] and locomotor activity [9] between the dLAN and control groups of mice. Borniger et al. [44] described this phenomenon as “internal desynchrony” between the central circadian oscillator in the suprachiasmatic nuclei and peripheral biological clocks. The external light/dark information can directly affect the metabolic patterns of mice through the regulation of neuroendocrine systems [48]. Bomiger et al. [49] also reported that light at night causes anxiety, alters metabolism, and causes weight gain in rodents. 

Another question that remained is the altered sleep, thus we checked the expression of the sleep gene *PER1* under different conditions. *PER1* is a versatile gene. It plays multiple roles in an organism. Reports exist showing that the expression of the *PER1* gene becomes modulated during sleep deprivation [23]. Familial advanced sleep-phase syndrome (FASP), associated with the deletion PER gene, is a condition in which a person sleeps and wakes up early [50]. Similarly, the *PER* mutant exhibited reduced total sleep time in mice compared to WT mice [51]. Panagiotou and Deboer [19] also stated that chronic dLAN exposure disrupts normal sleep behavior and deteriorates overall health in young and aged mice. Moreover, the *PER1* gene is linked with anxiety, as well. Lavebratt et al. [24] showed that *PER* genetic variants are more vulnerable to stress in animals and blocking this gene protected against anxiety and stress. Studies on animals showed that mice knockout for *PER* exhibited reduced immobility as compared to WT mice in the forced swimming test, used to screen levels of stress and anxiety [52]. The reason given for this regards high dopamine levels. Other studies also suggested that dopamine metabolism is influenced by the *PER* gene, which in turn influences mood-related behaviors [52]. Increased levels of *PER* may cause reduced dopamine levels, leading to stress and anxiety. As we saw in our experiment, dLAN causes anxiety behavior and reduces the expression of *PER1*. Similar results were observed in mice subjected to chronic unpredictable stress as the expression of the *PER1* gene was reduced and the animals exhibited depression-like behavior [53]. The introduction of curcumin increases *PER1* expression and improves stress and anxiety conditions.

Studies have also shown the role of the *PER1* gene in the feeding behavior of mice. Laboratory animals show food anticipatory activity (FAA) behavior, which elevates locomotor activity during daily mealtime under the normal circadian scheme. This activity is absent in mice with a mutant *PER* gene [22]. The correlation between *PER1* and food anticipation is also explained in other studies. If feeding is restricted, it causes a change in the *PER1* expression in the brain [54]. The levels of *PER* expression are shown to be maximum during mealtime [55]. 

In the present study, the expression of the *PER1* gene became deleteriously altered after the dLAN and DD exposure. The expression fold change of hippocampal *PER1* mRNA was found to be decreased in both groups. In our earlier study (43), we analyzed the effects of curcumin on the *PER1* mRNA expression in a light/dark (LD) control group, in which we found that curcumin causes a remarkable elevation in the expression of the hippocampal *PER1* gene in the LD control group. We included a comparative assessment of the effect of curcumin on the LD control and the aberrant lighting (dLAN and DD) on *PER1* expression. However, there was a lack of supportive data in this area to conclude this hypothesis and it mat open new doors for future research.

The limitations of the present study concern the duration of the experiment and the lack of inclusion of a positive control (a proven anxiolytic compound). In future studies, an increased duration of the experiment and inclusion of a proven anxiolytic agent will provide more significant data, allowing for a better correlation. The other important aspect is that if curcumin is useful in the regulation of circadian rhythm, its effect may be synergized using other plant-based formulations. In this context, several well-known immunomodulators may prove to be promising candidates. These may include *Ocimum tenuiflorum, Withania somnifera, Tinospora cordifolia* and *Gymnema sylvestre, Phyllanthus emblica*, and *Syzygium cumini.* Synergizing the effect of curcumin with other herbal formulations would entail empirical optimization, thus opening up novel avenues of research in this area. Fortification of curcumin may also be explored with sea buckthorn seeds or with other parts of this plant. Regardless of these alluring possibilities, the present study is still important in broadening the overall use of curcumin in the context of human health. 

## 6. Conclusions

Overall, our results establish that exposure to dLAN changes body weight and induces anxiety-like behavior in mice. Although curcumin does not show any recuperational effect on the altered percentage of the weight gain, nonetheless, it does restore and improve the emotional behavior in dLAN and DD-exposed mice. Thus, the present work seems to be highly reassuring.

## Figures and Tables

**Table 1:** Experimental design (*n* = 5) mice in each group.

S. Number	Groups	Experiment Conditions	Treatment	Duration
1	Light/Dark (LD) control	12 h:12 h light/dark [~150 lux]/[~0 lux]	1% CMC	3 weeks
2	Dim Light at Night (dLAN) control	12 h:12 h light/dim light [~150 lux]/[~5 lux]	1% CMC	3 weeks
3	Complete Darkness (DD) control	12 h:12 h dark/dark [~0 lux]/[~0 lux]	1% CMC	3 weeks
4	dLAN + Cur50	12 h:12 h light/dim light [~150 lux]/[~5 lux]	50 mg/kg curcumin	3 weeks
5	dLAN + Cur100	12 h:12 h light/dim light [~150 lux]/[~5 lux]	100 mg/kg curcumin	3 weeks
6	dLAN + Cur150	12 h:12 h light/dim light [~150 lux]/[~5 lux]	150 mg/kg curcumin	3 weeks
7	DD + Cur50	12 h:12 h dark/dark [~0 lux]/[~0 lux]	50 mg/kg curcumin	3 weeks
8	DD + Cur100	12 h:12 h dark/dark [~0 lux]/[~0 lux]	100 mg/kg curcumin	3 weeks
9	DD + Cur150	12 h:12 h dark/dark [~0 lux]/[~0 lux]	150 mg/kg curcumin	3 weeks

**Table 2:** Quantitative/real-time PCR primer pairs.

S. Number	Gene	Forward Primer (5′-3′)	Reverse Primer (5′-3′)
1	*PER1*	TTGGCAGGCTTCGTGGACTTG	GCGGGAACGCTTTGCTTTAGAT
2	*β*-*actin*	TGGTGGGTATGGGTCAGAAGGACTC	CATGGCTGGGGTGTTGAAGGTCTCA

## Data Availability

Data presented in this study are available on request from corresponding author.
